# Influence of Railway Traveling upon Public Health

**Published:** 1862-06

**Authors:** 


### INFLUENCE OF RAILWAY TRAVELLING UPON PUBLIC HEALTH.

*Extract from Report of British Commission.*

Physiological Influence.—*Habitual travelling.*—In considering the physiological influence of railway travelling upon public health, it is necessary to enlarge the sphere of observation beyond the mere act of travelling, and to regard some of the accessory conditions which surround it. Modern society has seized the occasion offered by increased facilities for business communication, and multiplied its means of pleasure and chances of recreation. Habitual railway travellers are found not only amongst those who move from place to place, urged by some necessary impulse; but also there is included amongst them a numerous class of season-ticket holders who, from considerations of health, pleasure, or economy, locate their families at a distance, and daily travel by train to some great industrial centre; again seeking a country home in the evening. This most important class of travellers is especially subject to the extraneous influence alluded to. They are under the necessity of twice daily 'catching the train;' and to a certain extent all their actions prior to departure in the morning and afternoon are influenced by the pervading sense of this anxiety. We will quote here a graphic communication on this matter from Dr. Forbes Winslow.

*Dr. F. Winslow on extraneous sources of anxiety and fatigue.*—"I have, like many others, during the summer season, removed my family for a period to a watering-place some fifty miles from London, and travelled to and fro night and morning by express train. I have been convinced that the advantage of sleeping by the sea-side, and of an occasional day of rest there, was fully counterbalanced by the fatigue and wear and tear of mind and body incidental to daily journeys over this considerable distance. I went to bed at night conscious that I must rise at a given and somewhat early hour, or miss my train. I am sure that this does not render sleep more sound and refreshing; and every one sleeps best on the Saturday night; when this disturbing element does not exist—since the next is the day of rest. In the same way breakfast is eaten with this necessity of being in time still on one's mind. Then, like every one else, I had to get the cab or carriage and go down to the station; to scramble for the morning paper and get a seat. Then comes the long journey, with all its fatiguing accompaniments. Finally, one has to get to one's residence; this process, or something like it, *mutatis mutandis*, being repeated twice a day. I refer to these separate details because it is in analyzing the general series of phenomena that I am able to explain the fatiguing effects, mental and physical, of constant railway travelling."

*Concurrent testimony of other physicians.*—The frequent ill-effects of this anxiety and mental pre-occupation are insisted on in the communications made to us by many physicians of great experience. They have a most important bearing in relation to certain diseases which will be elucidated in a subsequent section. Dr. Brown-Sequard observes, that the anxiety is so predominant in many otherwise healthy constitutions, that it produces often a practical incapacity for habitual travelling; and must evidently, when it falls short of this, frequently be injurious. When living near a railway station, he observed that the frequent excitement, and constant hurry and anxiety which he witnessed on the part of passengers arriving, impressed itself on his mind as a mischievous circumstance. In certain unhealthy conditions of the heart it has many times proved fatal.

But the actual fatigue and physical effects of the journey chiefly influence the mass; phlegmatic and robust-minded persons are little likely to allow themselves to be mentally agitated by the necessity for catching trains or the like. But let it here be noted, that a great number of the season-ticket travellers are men past the early vigor of life—time-worn by the continued cares of business during long residence in towns, and whom a more or less tardy success in the battle of life has enabled to afford for their families the salutary change of country or sea-side residence, or whom some warning of failing health compels to seek for themselves such a transformed version of the *rus in urbe.* This is a sort of constitution not able to bear unnecessary strain and fatigue.

We will next consider what are the conditions inducing functional change to which travellers are subjected in a railway train, proceeding at ordinary speed.

*Effects on the muscular system.*—The immediate effect of being placed in a vehicle subjected to rapid, short vibrations and oscillations, as already detailed, is that a considerable number of muscles are called into action, and maintained in a condition of alternating contractile effort throughout the whole journey. The tendency of each movement is to produce more or less motion in the twenty-four pieces of which the spine is made up. These movements are counteracted, and the erect position of the body is maintained, by the adoptive construction of that complex muscular system attached to those osseous pieces. The more violent movements of the carriage call into action the various sets of muscles in the back and chest; and it is only by an incessantly varying play of contraction and relaxation that the body is preserved in a tolerable state of equilibrium, and that the passenger combats with success the tendency to be shaken into a most unpleasing variety of shapes and positions. The head is especially thus affected, being so balanced on the spine as to have a tendency to fall forward.

The frequency, rapidity, and peculiar abruptness of the motion of the railway carriage keep thus a constant strain on the muscles; and to this must be ascribed a part of that sense of bodily fatigue, almost amounting to soreness, which is felt after a long journey.

*Influence on the cerebral and spinal centres.*—The hollow cavities of the spine and cranium, thus partially steadied, by the muscle attached to them contain the great nervous centres, to which concussion of any kind so peculiarly hurtful that they are naturally cushioned on exquisitely-devised waterbeds or are slung by fibrous ligaments, which have the effect of deadening the shocks of ordinary movements. It is unnecessary to dwell upon the injurious effects of commotion of the brain or spinal system of nerves. Cerebral or spinal concussions, in their higher degree, annihilate the functions of those organs. In the milder forms they lead up to disease which, remaining for a long time latent, may still end in paralysis.

The jolting of a railway carriage is a series of small and rapid concussions; and it is worthy to be noted that these increase in proportion to the rate of speed, and that most of the trains by which season-ticket holders travel are express. It may therefore be judged, so far as anatomical grounds are concerned, how prejudical must be the influence of reiteral concussions.

*The seats of carriages.*—The well-padded and springy seats of first-class carriages do much to obviate the mischief of these concussions for those who can afford to travel by them; and it has been mentioned to us by a surgeon who, in a great manufacturing centre, is connected officially with several railway companies, that he has observed that men compelled to travel frequently by rail are fully sensible of the practical advantage thus offered by the padded or cushioned seats, and never, if they can avoid it, travel on any other. We believe that commercial travellers, as a rule, occupy first-class carriages, not as a luxury, but as a necessity of their nomadic life. But however this may be, it is right to notice a remarkable fact in the relation of different classes of railway travellers. In 1849 the third-class passengers formed about 51 per cent. of the whole number; in 1860 they formed more than 57 per cent. It therefore becomes a matter of simple justice that the railway companies consider the health of this important class of travellers. In condemning them to sit on hard wooden benches, which transmit fully the shocks incidental to the movement of the carriage, they submit them unnecessarily to one acknowledged source of evil influence on health.

The influence of railway travelling upon the brain cannot, however, be measured solely by estimating the character and extent of the concussions to which it is subjected. The brain is not only affected by the mind, but also through the avenues of the eye and ear; and by the excitement of the respiratory and circulatory systems.

*Of mental influences.*—The mental condition of passengers by train is commonly, perhaps, sufficiently placid and unconcerned; but several eminently careful observers have, in their communications, alluded to an often experienced condition of uneasiness, scarcely amounting to actual fear, which pervades the generality of travellers by rail. The possibility of collision is constantly present to such persons. And every one knows how, if by chance a train stop at some unusual place, or if the pace be slackened, or the whistle sound its shrill alarm, a head is projected from nearly every window, and anxious eyes are on the look-out for signs and danger. So, too, the frequent lateness of trains, and the bad time which they keep, are causes of anxiety. The pace, also, prevents the traveller from observing natural objects and sites of interest on the road, which made coach travelling a source of mental relaxation and a pastime. The passenger is forced into subjective sources of mental activity; and where the tendency to excitement exists, this also, *quantum valeat*, must be esteemed an undesirable feature belonging to this manner of locomotion.

*Effect on the eye.*—Objects on the road are passed with such velocity that they only produce momentary impressions on the retina; and thus the visual powers are severely tried. The rapidity with which the brain is necessitated to take cognizance of the retinal images taxes it also more or less heavily. The rapid impression of objects moving over the retina in railway travelling is productive of certain interesting physiological phenomena, to which we can only briefly allude. Sir David Brewster has touched upon some of them with the ability for which he is distinguished. In a paper read at the last meeting of the British Association, and which will doubtless be published in the forthcoming volume of the "Proceedings," this great physicist showed that "when we look at the lines which stones, gravel, or other objects form in consequence of the deviation of their impressions on the retina, and quickly transfer the same lines further back, where the velocity is less, the stones or gravel or other objects would for an instant be distinctly seen, just as we see distinctly rapidly revolving objects in the dark when they are illuminated by an electric flash, or the light of an exploded copper cap." This phenomenon may be explained by a remarkable and interesting law of compensation. The rapidity and variety of the impressions necessarily fatigue both the eye and the brain. The constantly varying distance at which the objects are placed involves an incessant shifting of the adaptive apparatus by which they are focused upon the retina; and the mental effort by which the brain takes cognizance of them is scarcely less productive of cerebral wear because it is unconscious; for no fact in physiology is more clearly established than that excessive functional activity always implies destruction of material and organic change of substance. Dr. Budd, F. R. S., has directed our attention to the peculiarly dazzling effect of passing in rapid succession the white telegraph posts, from which the wires seem to fall and rise, appearing to the eye to undulate. When the traveller sets himself to read, he imposes yet further labor on the eye in tracing the shifting characters of his book or newspaper, and also on the brain. Especial notice is drawn to this point in an important communication which we have received, and shall hereafter publish in full, from Mr. White Cooper, surgeon-oculist to the Queen. Every one is conscious of the strain upon eyesigt incidental to railway travelling and to reading in the train; but it is probable that few habitual travellers are sufficiently cautious in this matter, and that many only become wise by too tardy an interpretation of their personal experience.

*Impressions through the ear.*—The rattle and noise which accompany the progress of the train create an incessant vibration on the tympanum, and thus influence the brain through the nerve of hearing. Noise is conveyed by vibrations of the air; and hearing is the transmission to the brain, through the medium of the auditory nerve, of those measured vibrations which constitute special sounds. The habitual traveller is little mindful of it, and the brain of many persons is perfectly tolerant of the excitement. But this is an important item in the general cerebral impressions produced. Dr. Fuller of St. George's Hospital, in communicating his personal experience of the influence of habitual railway travelling, mentions a device adopted by him which furnishes at once a test and remedy for this source of cerebral excitement. Surmising that some part of the headache and mental fatigue experienced was due to the rattle and noise, he filled each ear with a light plug of cotton-wool. In this way the vibrations of the air in the meatus were arrested, and the relief is described as having been considerable.

Assailed through the avenues of the eye and ear, and subject to concussions due to vertical movement and lateral oscillation communicated through the trunk, and actually transmitted by the bony walls of the head when it rests against the back of the carriage, the brain is apt to suffer certain physiological changes. Amongst the well-known effects are—occasional dizziness, headache, sickness, and mental fatigue.

*Combined effects on the brain.*—To these results each of the above causes may contribute something. The noise may be considered most influential in producing headache; but this may be added by the impression of rapidly-passing objects on the eye, or by the effort of reading. For nothing tends more certainly to produce congestion of the base of the brain than the forced fixation of the eyes upon a near object. This is evidenced by experiment, and explained by the connexion of the circulation of the eye with that of the brain, through the ophthalmic artery, springing from the circle of Willis. Some powerful illustrations of this fact are afforded in the phenomena of hypnotism and electro-biology, and of some of the so-called mesmeric phenomena, where sleep and the required changes of the cerebral circulation are physically produced by fixing the eye upon an adjacent shining body. The mere aspect of a swinging object sometimes suffices to produce sickness, as in the late celebrated Dr. Jas. Johnson; and so rapidly-passing objects may aid in the frequent production of nausea in the train. Probably the concussion incidental to the movement of the train is more potential in bringing about this effect. The influences on the respiration and circulation will hereafter be mentioned.

Any sudden alteration of the heart's action may aid in producing giddiness and dizziness, which are not always felt during the journey so strongly as after leaving the carriage. And it is when the circulation is returning to a more regular standard that temporary access of sickness and vertigo is often experienced. These symptoms have been especially noticed in the case of the travelling Post-office officials, as to whom Dr. Waller Lewis, the medical superintendent of the Post-office, has communicated to us a most valuable mass of information, which will appear in the third Report.

*Nausea and sickness.*—The tendency to nausea sometimes felt by railway travellers, without any apparent complication of cerebral disturbance, is often due to jolting of the intestines and gallbladder, stimulating the latter to discharge its contents into the stomach, and thus excite biliary sickness. Many cases have been communicated to us of sickness induced by railway travelling, and some persons are unable to undertake a journey without providing against this unpleasant affection. Bilious subjects Dr. Walter Lewis considers, from his experience, to be unfit for the travelling service, and he rejects such candidates for that employment.

*The nervous influences.*—In the valuable communications on the subject of this inquiry made to us by Dr. Brown-Sequard, the probable cause of sickness is explained by reference to the influence of the movements in a railway carriage on the phrenic, splanchnic, and vagus nerves. It is known by the experiments of several physiologists, that irritation of the vagus nerve produces vomiting; and the jolting of the stomach, separating it from the diaphragm and producing corresponding traction of the œsophagus, (which is attached to the diaphragm, when passing through that muscular septum,) is admitted by physiologists to afford the best explanation of the ordinary phenomena of sea-sickness. A second cause of vomiting, as shown by Luschka, is the irritation of the phrenic nerves due to the shaking of the diaphragm. Dr. Brown-Sequard, in former publications, has already investigated this subject experimentally, and has not only given confirmation to these observations, but has carried them further; and the result of his experiments will serve to give the physiological explanation of the peculiar *faintness* always accompanying nausea arising from the movements of a ship or railway carriage. He has shown that irritation of the sympathetic nerve in the abdomen induces cardiac syncope by a reflex action, influencing the action of the heart through the impressions conveyed by the splanchnic nerves acting upon the vagus through the spinal cord and medulla oblongata. The reflex character of the phenomena can be demonstrated by dividing the vagus in animals, when the irritation of the sympathetic no longer produces this cessation of the heart's beat, or by the division of the spinal cord between the communication of the sympathetic and the origin of the vagus, when a similarly negative effect is produced. Thus the faintness which sometimes occurs to travellers in a railway carriage, and the sickness accompanied by faintness, equally find their true scientific explanation. Every one has observed the marble pallor which accompanies this nausea; it is simple the result of a true fainting of the heart always preceding this form of sickness. It is needless to insist upon the important application of this physiological fact to all those persons who, being the subjects of fatty degeneration or other disease of the heart, are most likely to suffer from this kind of syncope, and in whom the organ may less readily recover from the temporary paralysis. There are two remedies of great value in diminishing these evil effects. A thoroughly tight bandage around the abdomen; inasmuch as it prevents, to some extent, the shaking of the stomach, and consequent irritation of the vagus nerve, so precluding the occurrence of sickness; and chloroform taken into the stomach may similarly prevent or diminish the annoyance by partially paralysing the vagus. —*London Lancet.*

